# Biological Impact and Enzyme Activities of *Spodoptera litura* (Lepidoptera: Noctuidae) in Response to Synergistic Action of Matrine and *Beauveria brongniartii*

**DOI:** 10.3389/fphys.2020.584405

**Published:** 2020-11-02

**Authors:** Jianhui Wu, Jiaying Li, Can Zhang, Xintong Yu, Andrew G. S. Cuthbertson, Shaukat Ali

**Affiliations:** ^1^ Key Laboratory of Bio-Pesticide Innovation and Application, Engineering Research Centre of Biological Control, South China Agricultural University, Guangzhou, China; ^2^ Yongzhou Tobacco Company, Yongzhou, Hunan, China; ^3^ Department of Eco-Engineering, Guangdong Eco-Engineering Polytechnic, Guangzhou, China; ^4^ Independent Researcher, York, United Kingdom

**Keywords:** *Spodoptera litura*, matrine, *Beauveria brongniartii*, antioxidant enzymes, nutritional efficiency

## Abstract

Matrine, a naturally occurring heterocyclic compound, has been shown to enhance the pathogenicity of the entomopathogenic fungus *Beauveria brongniartii* against *Spodoptera litura*. In the current study, the biological impacts and synergism activities of these two agents on nutritional efficiency and antioxidant enzymes in *S. litura* were explored. Our results showed a high antifeedant activity of *B. brongniartii* and matrine on *S. litura*. The *S. litura* larvae were unable to pupate and emerge when treated with combinations of matrine and *B. brongniartii*. Following on, we measured the activities of five important antioxidant enzymes [superoxide dismutase (SOD), peroxidase (POD), catalase (CAT), acetylcholinesterase (AChE), and glutathione-S-transferase (GST)] when treated with *B. brongniartii* SB010 (1 × 10^9^ spores/ml), matrine (0.5 mg/ml), and *B. brongniartii* SB010 (1 × 10^9^ spores/ml) + matrine (0.5 mg/ml). The results indicated the detoxification activity of the five enzymes in the fat body and hemolymph of *S. litura* when facing a combined *B. brongniartii* and matrine challenge. The activities of the enzymes were significantly lower than that of the control group 7 days post-treatment, indicating the inhibitory effect of the two xenobiotics. Matrine had better inhibition effects than *B. brongniartii* in a majority of the trials. The improved detoxification activity of the five enzymes may be the internal mechanism of synergism of matrine on *B. brongniartii*.

## Introduction

The tobacco cutworm, *Spodoptera litura* (Fabricius; Lepidoptera: Noctuidae), is a serious polyphagous herbivore, which has caused significant economic damage to cropping systems around the world. As a result, over the past decades large quantities of chemical pesticides have been applied for the control of *S. litura*. However, overuse of synthetic pesticides has resulted in reports of adverse effects, such as pesticide resistance and pest resurgence, and also the contribution to negative impacts on human health and other components of the environment (Cuthbertson and Murchie, 2005; Ishtiaq et al., 2012; Ahmad and Mehmood, 2015). Due to these reasons, finding alternate control methods including the use of natural control agents is continuing to play an important role in developing management strategies for *S. litura* (Revathi et al., 2014).

Entomogenous fungi are important agents for the biological control of *S. litura* (Chaudhari et al., 2016; Nguyen et al., 2017; Yang et al., 2019). As one of the most common insect pathogenic fungal species, fungi belonging to the genus *Beauveria* (Hypocreales: Cordycipitaceae) have shown high insecticidal activity against *S. litura* (Borja et al., 2018; Dhar et al., 2019; Yang et al., 2019). Among them, *Beauveria brongniartii* is one of the most effective pathogenic species and has been widely used in insect control (Fan et al., 2013; Liu et al., 2016). Previous studies have shown that *B. brongniartii* isolate SB010 has high pathogenic activity against different insect species (Huang, 2019; Ou, 2019).

Matrine, a naturally occurring heterocyclic compound, is isolated from the roots of *Sophora flavescens* (Kushen) and *Sophora alopecuroides* (Fabales: Fabaceae; Mao and Henderson, 2007; Zhang et al., 2012; Cheng et al., 2018). Since matrine was discovered, its biological activity has received worldwide attention. It has been shown that matrine has a wide range of biological properties including antitumor activity, antiviral activity, and antifungal activity (Li et al., 2010; Liu et al., 2014; Zanardi et al., 2015). As a well-known traditional medicine in China, matrine has also been shown to have broad spectrum insecticidal activity, as well as being relatively non-toxic to the environment (Huang and Xu, 2016; Ali et al., 2017). Based on the research of Wu et al. (2019), matrine could significantly increase the pathogenicity of *B. brongniartii* isolate SB010 and as such is considered as a potential control agent against *S. litura*.

Insects are known to cope with threats of both insecticide and pathogen through the action of different enzyme systems (Terriere, 1984; Roslavtseva et al., 1993; Serebrov et al., 2006; Dubovskiy et al., 2012). General esterases (ESTs), glutathione-S-transferase (GST), and multi-function oxidases (MFOs) are most commonly involved in insect defense (Li et al., 2007). MFOs involved in the P450 pathways are involved in detoxification of natural and synthetic xenobiotics (Feyereisen, 2005). GST plays a pivotal role in detoxification and cellular antioxidant defenses against oxidative stress by conjugating reduced glutathione to the electrophilic centers of natural and synthetic exogenous xenobiotics, including insecticides, allelochemicals, and endogenously activated compounds (Ortelli et al., 2003; Enayati et al., 2005; Lumjuan et al., 2005). ESTs play an important role in insect defense through catabolism of the esters of higher fatty acids that influence flight as well in the degradation of inert metabolic esters (Terriere, 1984; Roslavtseva et al., 1993) and various xenobiotics, including insecticides. Changes in the activities of these enzymes are reflected in insect resistance to insecticides as well as in degradation of toxic molecules produced during *M. anisopliae* infection and, therefore, play a key role in the protection of insects against pathogens (Terriere, 1984; Roslavtseva et al., 1993; Serebrov et al., 2006; Dubovskiy et al., 2012).

Several other insect enzymes [including antioxidant enzymes such as superoxide dismutase (SOD), catalase (CAT), and peroxidase (POD)] provide defenses against pathogens and insecticides (Felton and Summers, 1995). Previous studies showed that these enzymes can be quickly upregulated in response to xenobiotic threats and that increase in the activities of these enzymes are related to pesticide resistance and melanization in insects (Campa-Córdova et al., 2002; Müller et al., 2007; Gopalakrishnan et al., 2011; Wu et al., 2011). The phenol oxidase (PO) cascade protects insects from microbial infections through its involvement in the melanization of hemocytes attached to parasite surfaces (Cerenius and Söderhäll, 2004). Increased PO activity can improve immunity to xenobiotics and promote wound healing (Liu et al., 2009). Acetylcholinesterase (AChE) is a key enzyme catalyzing the hydrolysis of the neurotransmitter acetylcholine (Ach) in the nervous system of various organisms (Oehmichen and Besserer, 1982). AChE is affected by organophosphate and carbamate insecticides, botanical insecticides, and secondary fungal metabolites (Zibaee et al., 2009). Although previous experiments by Wu et al. (2019) showed that the combination of *B. brongniartii* and matrine had a synergistic effect; however, the specific antioxidant defense response in *S. litura* is not clear when facing a combined *B. brongniartii* and matrine challenge.

In this study, the biological impacts of *B. brongniartii* and matrine on nutritional efficiency and detoxification enzymes (GST, AChE, SOD, CAT, and POD) in *S. litura* were explored. Our results provide further information in support of the combined application of entomopathogenic fungi and natural chemicals in the development of *S. litura* control strategies.

## Materials and Methods

### Insects

Larvae of *S. litura* were reared on a semi-synthetic artificial diet prepared following the method of David et al. (1975) and kept under laboratory conditions of 26 ± 2°C, 65 ± 5% relative humidity (r.h.), and 16:8 h (light:dark) photoperiod at South China Agricultural University (SCAU), Guangzhou, China.

### Chemicals and Fungal Preparations

Matrine (95% purity) was obtained from Guangdong New Scene Bioengineering Company, Yangjiang, China. Five different concentrations of matrine (0.05, 0.125, 0.25, 0.5, and 1.0 mg/ml) were prepared by dissolving matrine powder in methanol.

*Beauveria brongniartii* isolate SB010 was used in the trials. This isolate was obtained from soil samples taken from the experimental farm fields of SCAU and kept in the Key Laboratory of Biopesticides Innovation and Application of Guangdong Province, SCAU. A fungal spore suspension (1 × 10^9^conidia/ml) was then prepared following the method of Ali et al. (2017).

## Scheme of Experiments

The efficacy and nutrition trails were conducted by applying nine different individual or combined treatments of *B. brongniartii* and matrine as outlined in Table 1. In case of enzymatic studies, four different individual or combined treatments of *B. brongniartii* and matrine as shown in Table 2 were applied to study the variations in enzymatic studies.

**Table 1:** Combinations of *Beauveria brongniartii* and matrine tested during nutrition analysis.

Treatment	*Beauveria brongniartii* (conidia/ml)	Matrine(mg/L)
T1	0	0
T2	0	0.5
T3	0	1.0
T4	1 × 10^8^	0
T5	1 × 10^9^	0
T6	1 × 10^8^	0.5
T7	1 × 10^8^	1.0
T8	1 × 10^9^	0.5
T9	1 × 10^9^	1.0

**Table 2:** Different treatments of *Beauveria brongniartii* and matrine applied during enzyme analysis.

Treatment	*Beauveria brongniartii* (conidia/ml)	Matrine (mg/L)
T1	0	0
T2	0	0.5
T3	1 × 10^9^	0
T4	1 × 10^9^	0.5

### Nutrition Analysis

Newly molted third instar larvae of *S. litura* from the same batch were used for the nutrition experiment. Individual or combined treatments of *B. brongniartii* and matrine as outlined in Table 1 were added during the molten state of artificial diet preparation. Artificial diet without the addition of fungal conidia or matrine designated as T1 in Table 1 served as control. The larvae (10 individuals) were individually placed in plastic cups (diameter: 3 cm and height: 4 cm) to feed on 1 g of pre-treated as well as control diet. The experimental setup was incubated at 25 ± 2°C, 80 ± 5% relative humidity, and 16:8 h (light:dark) photoperiod. The whole experimental setup was repeated thrice, thus each treatment was repeated 30 times. The larvae and artificial diet were weighed everyday by using an electronic balance (precision: 0.0001 g) to record the larvae weight gain and food weight loss until 21 days post application. The weight of the third instar larvae after 12 h of molting was used as the starting weight. The pupation rate and emergence rate were also recorded.

Nutritional indices were calculated according to Koul et al. (1997, 2004):

Relative growth rate (RGR) = (change in the larval dry weight per day/starting larval dry weight).

Relative consumption rate (RCR) = change in the larval diet dry weight per day/starting larval dry weight.

Efficiency of conversion of ingested food (ECI) = 100 × dry weight gain of the insect/dry weight of food consumed.

The number of successfully pupated *S. litura* larvae treated with individual or combined treatments of *B. brongniartii* and matrine was recorded after 30 days of treatment. The rate of pupation (%) was calculated by following equation.

Percent pupation = (Number of pupated larvae/Total number of larvae) × 100.

The number of successfully emerged adults from *S. litura* larvae treated with individual or combined treatments of *B. brongniartii* and matrine was recorded after 45 days of treatment. The rate of emergence (%) was calculated by following equation.

Percent adult emergence = (Number of adults/Total number of pupae) × 100.

### Enzyme Analysis

Newly molted fourth instar larvae of *S. litura* were fed on a fresh artificial diet treated with a combined matrine and fungal conidia suspension as shown in Table 2. For control larvae, the worms were fed with artificial diet without the addition of fungal conidia or matrine. The treated larvae were placed in plastic cups followed by incubation at 26 ± 2°C and 65 ± 5% r.h. Following 3, 5, and 7 days, the samples (larvae) were collected and dissected to get access to their fat bodies and hemolymph. Each treatment consisted of three replicates and each replicate consisted of 10 larvae.

Fat body homogenates for enzyme assays were prepared by dissolving 0.6 g of fat bodies in 1.0 ml of specific buffers for each enzyme [0.05 mol/L Tris-HCL (pH 7.5) for GSTs assay; 0.02 mol/L PBS (pH 7.0) for AChE assay; 0.05 mol/L PBS (pH 7.8) for SOD assay; and 0.15 mol/L PBS (pH 7.0) for CAT assay; and 0.2 mol/L PBS (pH = 6.0) for POD assay] at 4°C followed by grinding with glass homogenizer on ice bath. The homogenates were centrifuged at 11,000 rpm for 10 min at 4°C, and the supernatant was taken as an enzyme source.

The hemolymph was sampled with a glass capillary through an incision in the cuticle and placed in cooled tubes to which 4 mg/ml phenylthiourea was added to prevent melanization. The hemolymph was centrifuged at 4°C for 10 min at 11,000 rpm, and the cell-free plasma fraction was used to determine the enzyme activity.

The GSTs were conducted using the procedures developed by Habig (1981). Incubation was carried out at 25°C for 5 min in 0.1 M Na-phosphate buffer (pH 6.5) containing 1 mM glutathione, 1 mM DNCB, and 20 μl of the sample. The reaction was initiated by adding DNCB solution in acetone. Concentration of 5-(2,4-dinitrophenyl) glutathione produced during the reaction was measured spectrophotometrically at the wavelength of 340 nm. One unit of enzyme will conjugate 10.0 nmol of CDNB with reduced glutathione per minute.

AChE activity was assayed by following Ellman et al. (1961) with some slight modifications. The reaction mixture contained 50 μl sample solution, 100 μl 45 μM 5-5-dithiobis-(2-nitrobenzoic acid), 100 μl acetylthiocholine iodide, and 90 μl sodium phosphate buffer. The change in absorbance at 405 nm was recorded for 40 min. One milliunit of AChE activity is the amount of enzyme that will generate 1.0 nmol of choline per minute.

SOD activity was measured in cell free extracts by nitro blue tetrazolium (NBT) reduction (Beauchamp and Fridovich, 1971). The assay mixture contained 0.2 ml 0.1M ethylene diamine tetraacetic acid (EDTA) containing 0.3 mM sodium cyanide (0.2 ml), 0.1 ml 1.5 mM nitroblue tetrazolium (NBT), 3 ml 0.067M potassium phosphate buffer, pH 7.8, and a series of samples ranging from 0.1 to 10 mg protein in different tubes. The tubes were placed in a light box providing uniform light intensity. The tubes were incubated for 5–8 min to achieve a standard temperature. At zero time, 0.05 ml 0.12 mM riboflavin was added and all the tubes were incubated for 12 min and absorbance was read at 560 nm after 1 min interval. One unit of SOD activity was defined as the amount of SOD required for inhibition of the reduction of NBT by 50% (A_560_) and was expressed as units per mg protein (U/mg protein).

CAT activity was assayed by the method described by Beers and Sizer (1952), in which the decomposition of H_2_O_2_ was analyzed spectrophotometrically at 240 nm. Reagent grade water (1.9 ml) and 0.059M hydrogen peroxide (1.0 ml) were pipetted into the cuvette. The cuvette was incubated in spectrophotometer for 4–5 min to achieve temperature equilibration and to establish blank rate if any. After 5 min, 0.1 ml of sample was added and change in absorbance at 240 nm was observed for 2–3 min. Change in absorbance per minute was calculated from the initial (45 s) linear portion of curve. One unit of CAT activity was defined as the amount of enzyme that decomposes 1 mmol H_2_O_2_/min at an initial H_2_O_2_ concentration of 30 mM at pH 7.0 and 25°C and was expressed as U/mg protein.

POD activity assay was performed by following Shannon et al. (1966). Briefly, 3.00 ml of reaction mixture having 2.1 ml H_2_O, 0.32 ml 14 mM potassium phosphate buffer, 0.16 ml 0.027% (v/v) hydrogen peroxide, and 0.32 ml 0.5% (w/v) pyrogallol was incubated 20°C for 10 min. The 0.1 ml of 14 mM potassium phosphate buffer and 0.1 ml of sample was added and followed by mixing through inversion. Change in absorbance was measured spectrophotometrically at the wavelength of 420 nm. Enzyme activity was expressed as U/mg protein.

The protein concentrations in the supernatants were determined by following the method of Bradford (1976) using bovine serum albumin as standard.

## Data Analysis

Data were subjected to one-way ANOVA and mean values were compared using Tukey’s HSD test (*p* < 0.05). All data analysis was performed using SPSS 19.0 software.

## Results

### Effect on the Feeding and Food Utilization Efficiency of *Spodoptera litura*

After feeding the *S. litura* larvae on the treated diets, nutritional analysis results showed significant differences in RGRs of *S. litura* in response to individual or combined treatment of *B. brongniartii* and matrine after 7 days (*F*_8,261_ = 45.77; *p* < 0.01), 15 days (*F*_8,261_ = 38.04; *p* < 0.01), and 21 days (*F*_8,261_ = 54.89; *p* < 0.01). The results of the RGR are shown in Table 3. Individual RGR values of *S. litura* larvae in combined treatments of *B. brongniartii* and matrine were significantly lower than those observed for individual treatments of *B. brongniartii* or matrine at different time intervals. The lowest RGR value of 0.05 ± 0.004 g/(g/day) was observed for T9 at different time intervals. Highest RGR values were observed for T1 (control) having mean values of 16.17 ± 2.37, 19.26 ± 0.54, and 23.93 ± 0.12 g/(g/day) after 7, 14 and 21 days of treatment, respectively. The RGR values observed for different combined treatments of *B. brongniartii* and matrine (T6–T8) were statistically at par with each other after 7, 14 and 21 days of treatment.

**Table 3:** Effect of *Beauveria brongniartii* and matrine on relative growth rate (RGR) of *Spodoptera litura*.

Treatment	RGR [g/(g/day)]		
	7 days	14 days	21 days
T1	16.17 ± 2.37a	19.26 ± 0.54a	23.93 ± 0.12a
T2	2.18 ± 0.03c	1.81 ± 0.40c	1.61 ± 0.04d
T3	2.02 ± 0.0005d	1.72 ± 0.03c	1.57 ± 0.05de
T4	8.48 ± 0.65b	7.55 ± 1.65b	5.55 ± 0.33b
T5	7.54 ± 0.16bc	7.07 ± 0.50b	5.23 ± 0.38bc
T6	1.13 ± 0.05c	1.13 ± 0.04c	0.81 ± 0.03e
T7	1.06 ± 0.04c	1.41 ± 0.02c	0.70 ± 0.04e
T8	1.41 ± 0.26c	1.23 ± 0.20c	0.37 ± 0.12e
T9	0.05 ± 0.004d	0.05 ± 0.004d	0.05 ± 0.004f

The data in the table are mean ± standard error. Following Tukey’s test, different lowercase letters indicate significant differences between different combinations of concentrations at the same time period (*p* < 0.05).

The analysis of RCR of *S. litura* in response to individual or combined treatment of *B. brongniartii* and matrine revealed significant differences after 7 days (*F*_8,261_ = 39.67; *p* < 0.01), 15 days (*F*_8,261_ = 41.23; *p* < 0.01), and 21 days (*F*_8,261_ = 69.51; *p* < 0.01) of application as shown in Table 4. Individual RCR values of *S. litura* larvae in combined treatments of *B. brongniartii* and matrine were significantly lower than those observed for individual treatments of *B. brongniartii* or matrine at different time intervals. The RCR values observed for different combined treatments of *B. brongniartii* and matrine (T7–T9) were statistically at par with each other after 7, 14, and 21 days of treatment. The lowest RCR values (0.82 ± 0.05, 1.00 ± 0.05, and 0.69 ± 0.05 g/(g/day) after 7, 14, and 21 days of treatment, respectively) were observed for T9 at different time intervals. Highest RCR values were observed for T1 (control) having mean values of 1.88 ± 0.02, 2.07 ± 0.05, and 2.29 ± 0.07 g/(g/day) after 7, 14, and 21 days of treatment, respectively. On the 7th day after treatment, the RCR value of *S. litura* in the T2 treatment was not significantly different from T1 (control). However, the other treatments were significantly lower than the control. Following 14 days after treatment, there was no significant difference among T1 (control), T2, and T3 treatments. However, the other treatments were significantly lower than T1 (control). On 21 days after treatment, the RCR values of treatment groups were significantly lower than the control group.

**Table 4:** Effect of *Beauveria brongniartii* and matrine on relative consumption rate (RCR) of *Spodoptera litura*.

Treatment	RCR [g/(g/day)]		
	7 days	14 days	21 days
T1	1.88 ± 0.02a	2.07 ± 0.05a	2.29 ± 0.07a
T2	1.74 ± 0.06a	2.03 ± 0.06a	2.17 ± 0.03a
T3	1.11 ± 0.04b	2.11 ± 0.04a	0.70 ± 0.01c
T4	0.94 ± 0.04bc	1.03 ± 0.03c	1.00 ± 0.04c
T5	1.07 ± 0.04bc	0.63 ± 0.02d	1.55 ± 0.05b
T6	1.03 ± 0.04bc	1.34 ± 0.04b	1.46 ± 0.05b
T7	0.93 ± 0.03bc	1.13 ± 0.05c	1.00 ± 0.02c
T8	0.93 ± 0.03bc	1.03 ± 0.04c	1.00 ± 0.04c
T9	0.82 ± 0.05c	1.00 ± 0.05c	0.69 ± 0.05c

The data in the table are mean ± standard error. Following Tukey’s test, different lowercase letters indicate significant differences between different combinations of concentrations at the same time period (*p* < 0.05).

The analysis of the ECI of *S. litura* in response to individual or combined treatment of *B. brongniartii* and matrine revealed significant differences after 7 days (*F*_8,261_ = 43.23; *p* < 0.01), 15 days (*F*_8,261_ = 45.89; *p* < 0.01), and 21 days (*F*_8,261_ = 63.31; *p* < 0.01) of application as shown in Table 5. Individual ECI values of *S. litura* larvae in combined treatments of *B. brongniartii* and matrine were significantly lower than those observed for individual treatments of *B. brongniartii* or matrine at different time intervals. The RCR values observed for different combined treatments of *B. brongniartii* and matrine (T6–T8) were statistically at par with each other after 14 and 21 days of treatment. The lowest ECI values (2.19 ± 0.01, 1.91 ± 0.01, and 1.23 ± 0.01% after 7, 14, and 21 days of treatment, respectively) were observed for T9. Highest ECI values were observed for T1 (control) having mean values of 65.47 ± 7.70, 34.02 ± 4.40, and 13.04 ± 0.61% after 7, 14, and 21 days of treatment, respectively. At three different time points, the ECI values of *S. litura* in the T4 treatment were not significantly different from that of the blank control (T1).

**Table 5:** Effects of *Beauveria brongniartii* and matrine on efficiency of conversion (ECI) of ingested food of *Spodoptera litura*.

Treatment	ECI (%)		
	7 days	14 days	21 days
T1	65.47 ± 7.70a	34.02 ± 4.40a	13.04 ± 0.61a
T2	15.61 ± 0.64b	21.64 ± 2.04b	6.60 ± 0.39b
T3	11.12 ± 0.001b	15.49 ± 0.16bc	3.46 ± 0.10b
T4	62.35 ± 9.17a	33.11 ± 3.12a	12.32 ± 0.87a
T5	22.47 ± 0.93b	28.75 ± 3.04a	10.3 ± 0.73a
T6	10.22 ± 0.72bc	6.26 ± 0.37c	4.13 ± 0.29b
T7	10.04 ± 0.71bc	5.63 ± 0.60c	4.6 ± 0.35b
T8	4.76 ± 0.34c	4.72 ± 0.28c	4.23 ± 0.17b
T9	2.19 ± 0.01d	1. 91 ± 0.01d	1.23 ± 0.01c

The data in the table are mean ± standard error. Following Tukey’s test, different lowercase letters indicate significant differences between different combinations of concentrations at the same time period (*p* < 0.05).

### Effects of *Beauveria brongniartii* and Matrine on Pupation and Emergence Rates in *Spodoptera litura*

**Figure 1:**
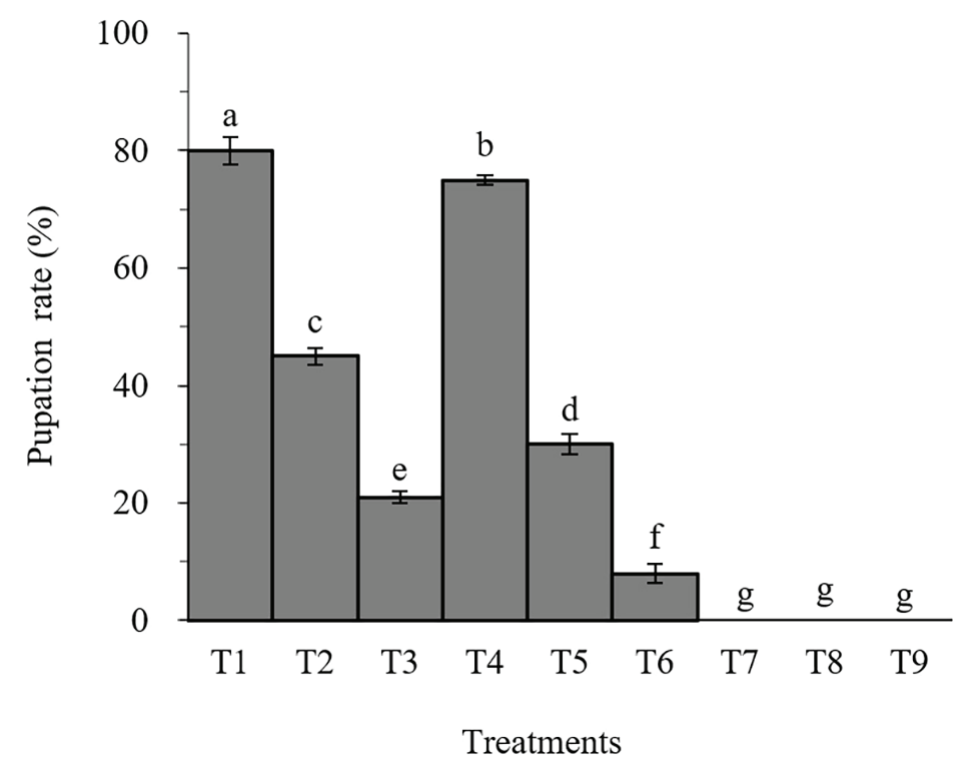
Effect of *Beauveria brongniartii* and matrine on the pupation rate of *Spodoptera litura*. Bars having different letters show significant differences between treatments. T1 = Control; T2 = Matrine 0.5 mg/L; T3 = Matrine 1.0 mg/L; T4 = *B. brongniartii* 1 × 10^8^ conidia/ml; T5 = *B. brongniartii* 1 × 10^8^ conidia/ml; T6 = *B. brongniartii11* 1 × 10^8^ conidia/ml + Matrine 0.5 mg/L; T7 = T6 = *B. brongniartii* 1 × 10^8^ conidia/ml + Matrine 1.0 mg/L; T8 = *B. brongniartii* 1 × 10^9^ conidia/ml + Matrine 0.5 mg/L; and T9 = *B. brongniartii* 1 × 10^9^ conidia/ml + Matrine 1.0 mg/L.

Percent pupation of *S. litura* larvae treated with individual or combined treatment of *B. brongniartii* and matrine differed significant among different treatments after 30 days of application (*F*_8,261_ = 31.07; *p* < 0.01). The effects of *B. brongniartii* and matrine on the pupation rate of are shown in Figure 1. The pupation rates of *S. litura* treated with *B. brongniartii* SB010 and matrine were significantly lower than the control (T1). When feed with combinations of SB010 and matrine (0.5 and 1.0 mg/L), the pupation rates were zero, except those in T6.

**Figure 2:**
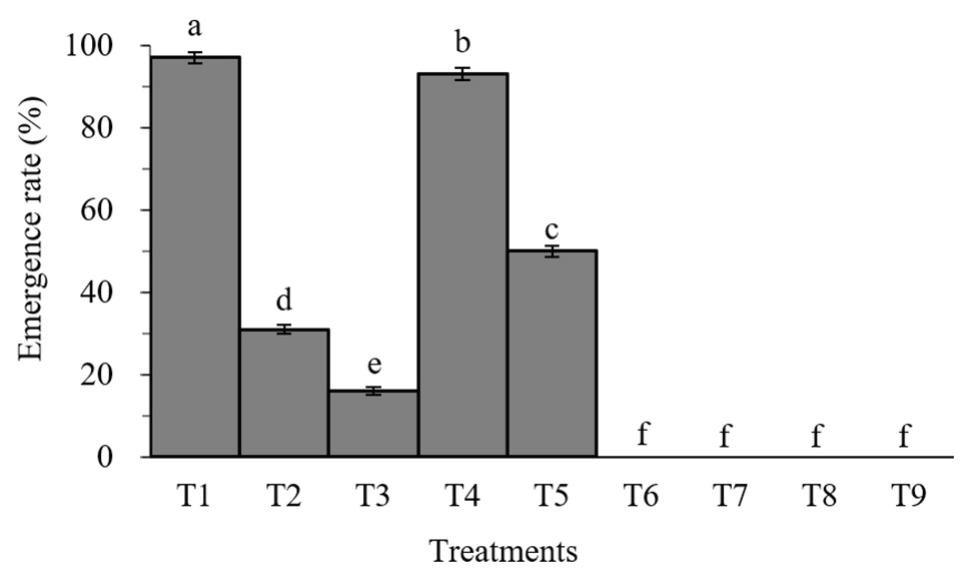
Effect of *Beauveria brongniartii* and matrine on the emergence rate of *Spodoptera litura.* Bars having different letters show significant differences between treatments. T1 = Control; T2 = Matrine 0.5 mg/L; T3 = Matrine 1.0 mg/L; T4 = *B. brongniartii* 1 × 10^8^ conidia/ml; T5 = *B. brongniartii* 1 × 10^8^ conidia/ml; T6 = *B. brongniartii* 1 × 10^8^ conidia/ml + Matrine 0.5 mg/L; T7 = *B. brongniartii* 1 × 10^8^ conidia/ml + Matrine 1.0 mg/L; T8 = *B. brongniartii* 1 × 10^9^ conidia/ml + Matrine 0.5 mg/L; and T9 = *B. brongniartii* 1 × 10^9^ conidia/ml + Matrine 1.0 mg/L.

Percent pupation of *S. litura* larvae treated with individual or combined treatment of *B. brongniartii* and matrine differed significant among different treatments after 45 days of application (*F*_8,261_ = 26.83; *p* < 0.01). As can be seen in Figure 2, the emergence rates of adult *S. litura* fed with *B. brongniartii* were significantly reduced compared to the control (T1). Also, the higher the concentration of fungal conidia and matrine, the higher the reduction in emergence rate. The results show that combined treatments of *B. brongniartii* and matrine can dramatically reduce the rate of emergence of *S. litura* to zero.

### Effects of *Beauveria brongniartii* and Matrine on the Enzyme Activities in *Spodoptera litura*

**Figure 3:**
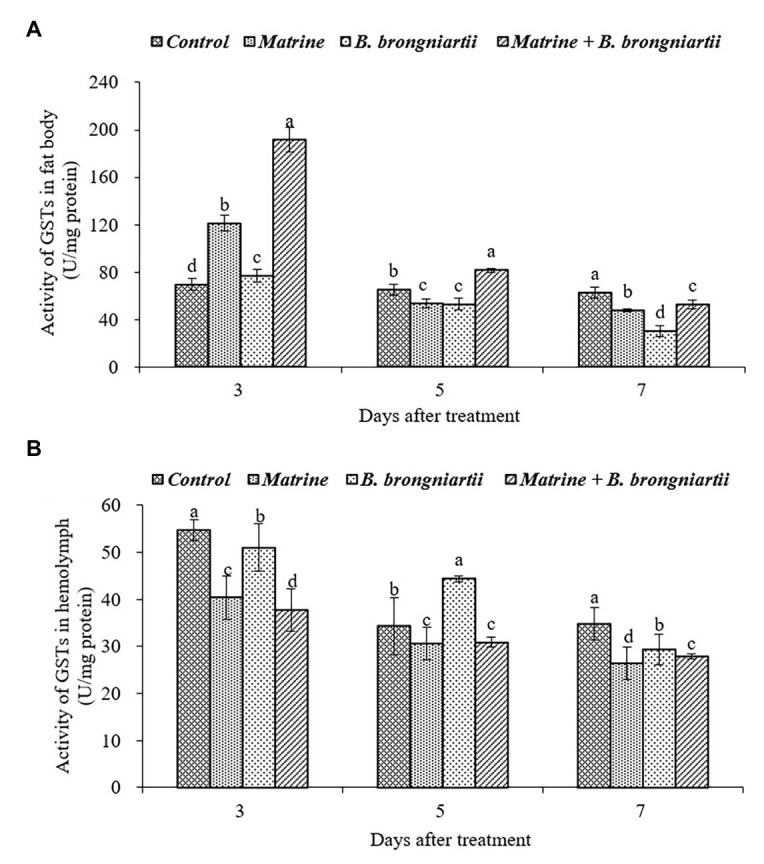
Changes in glutathione-S-transferase (GST) activity of *Spodoptera litura* following treatment with *Beauveria brongniartii* and matrine **(A)** in the fat body and **(B)** in hemolymph. Bars having different letters show significant differences between treatments at different time intervals.

After exposure of the fourth instar larvae to the treated diet, GST activity was also affected both in the fat body and in the hemolymph (Figure 3). In the fat body, the highest activity was found on the 3rd day following treatment by matrine and *B. brongniartii*. The lowest activity was found on the 7th day after being treated by SB010 (Figure 3A). In the hemolymph (Figure 3B), GST activity showed a decreasing trend and was significantly lower than the control in all cases except on the 5th day when treated by *B. brongniartii*.

**Figure 4:**
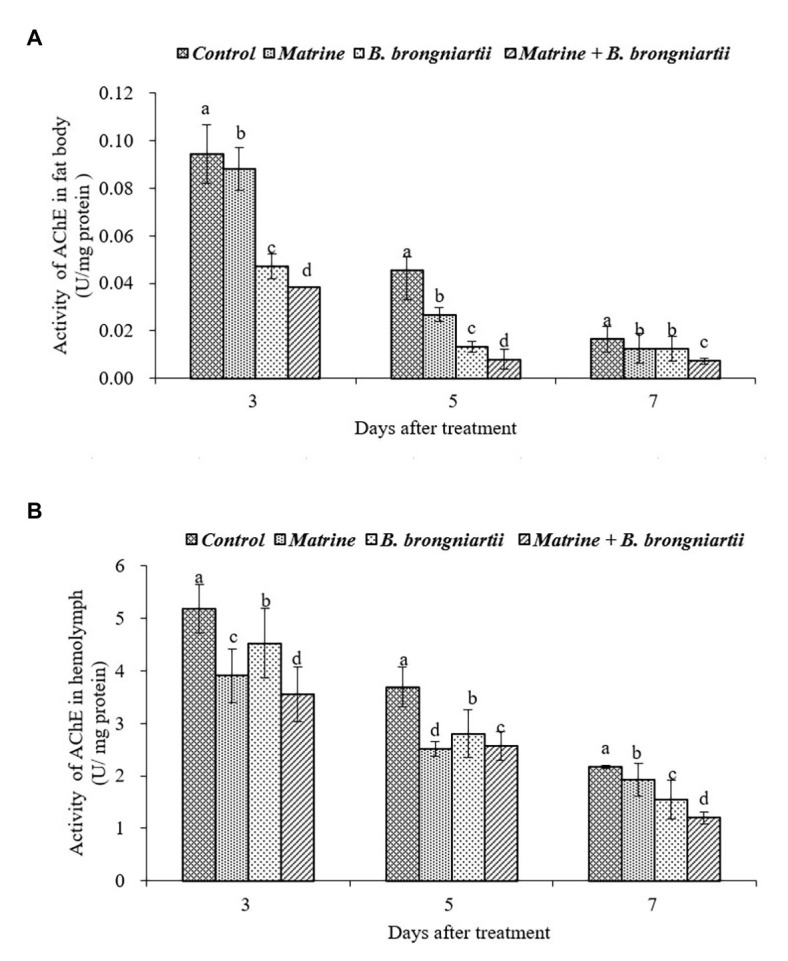
Changes in acetylcholinesterase (AChE) activity of *Spodoptera litura* after treatment with *Beauveria brongniartii* and matrine **(A)** in the fat body and **(B)** in hemolymph. Bars having different letters show significant differences between treatments at different time intervals.

The AChE activities in the fat body and in the hemolymph of *S. litura* showed the same trend (Figure 4). The results indicated that AChE activities decreased as time went by. The lowest activity in both the fat body and hemolymph was noted on the 7th day after feeding matrine and *B. brongniartii*. Activities of all treatments were significantly lower than the control.

**Figure 5:**
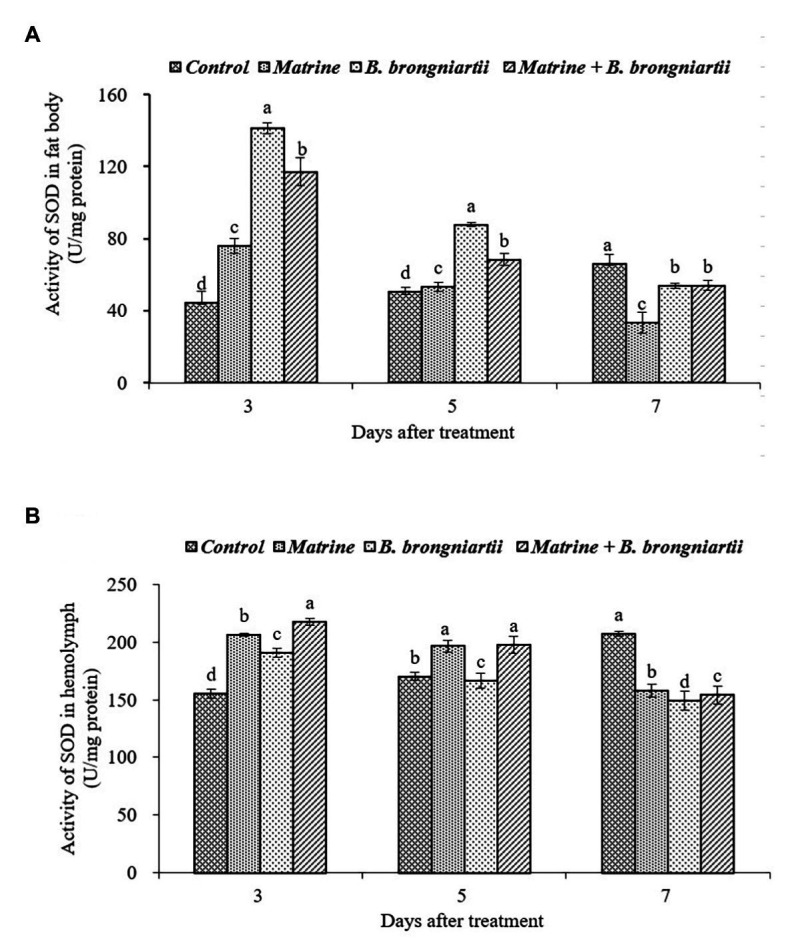
Changes in superoxide dismutase (SOD) activity of *Spodoptera litura* after treatment with *Beauveria brongniartii* and matrine **(A)** in the fat body and **(B)** in hemolymph. Bars having different letters show significant differences between treatments at different time intervals.

Following exposure of the fourth instar larvae of *S. litura* to treated diets with *B. brongniartii* and matrine, the enzyme activity of SOD differed significantly after 3, 5, and 7 days of feeding both in the fat body and in the hemolymph when compared to the control (Figure 5). The results showed that the activity of SOD following treatments was significantly different from that of the control. On days 3 and 5 after treatment, the activity of SOD in the fat body was significantly higher than that of the control (Figure 5A). The SOD activity then decreased significantly on day 7 compared to the control. The SOD activity in the hemolymph of the control showed an upward trend (Figure 5B). However, the values under different treatments decreased and showed a minimum activity 7 days post-treatment.

**Figure 6:**
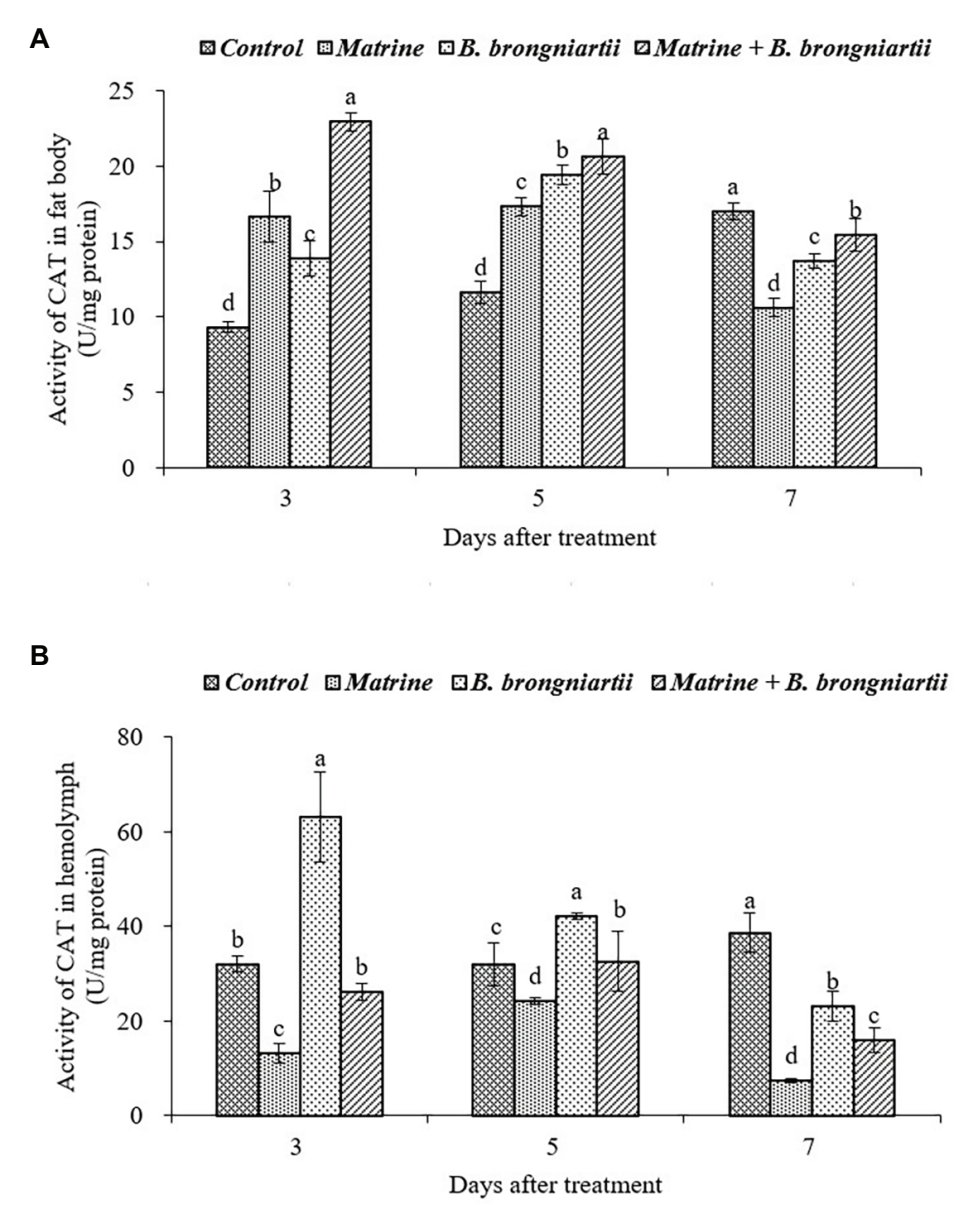
Changes in catalase (CAT) activity of *Spodoptera litura* following treatment with *Beauveria brongniartii* and matrine **(A)** in the fat body and **(B)** in hemolymph. Bars having different letters show significant differences between treatments at different time intervals.

As can be seen in Figure 6, feeding *B. brongniartii* and matrine could significantly affect the activity of CAT. On the 3rd and 5th days after treatment, CAT activity in the fat body showed an increasing trend and was significantly higher than the control after treatment by matrine, *B. brongniartii* and their combination. In comparison, the CAT activity decreased and was significantly lower in the control (Figure 6A). In the hemolymph (Figure 6B), only the CAT activity of *S. litura* feeding with matrine increased on the 3rd and 5th days following treatment. On the 7th day after treatment, CAT activity showed a decrease both in the fat body and in the hemolymph.

**Figure 7:**
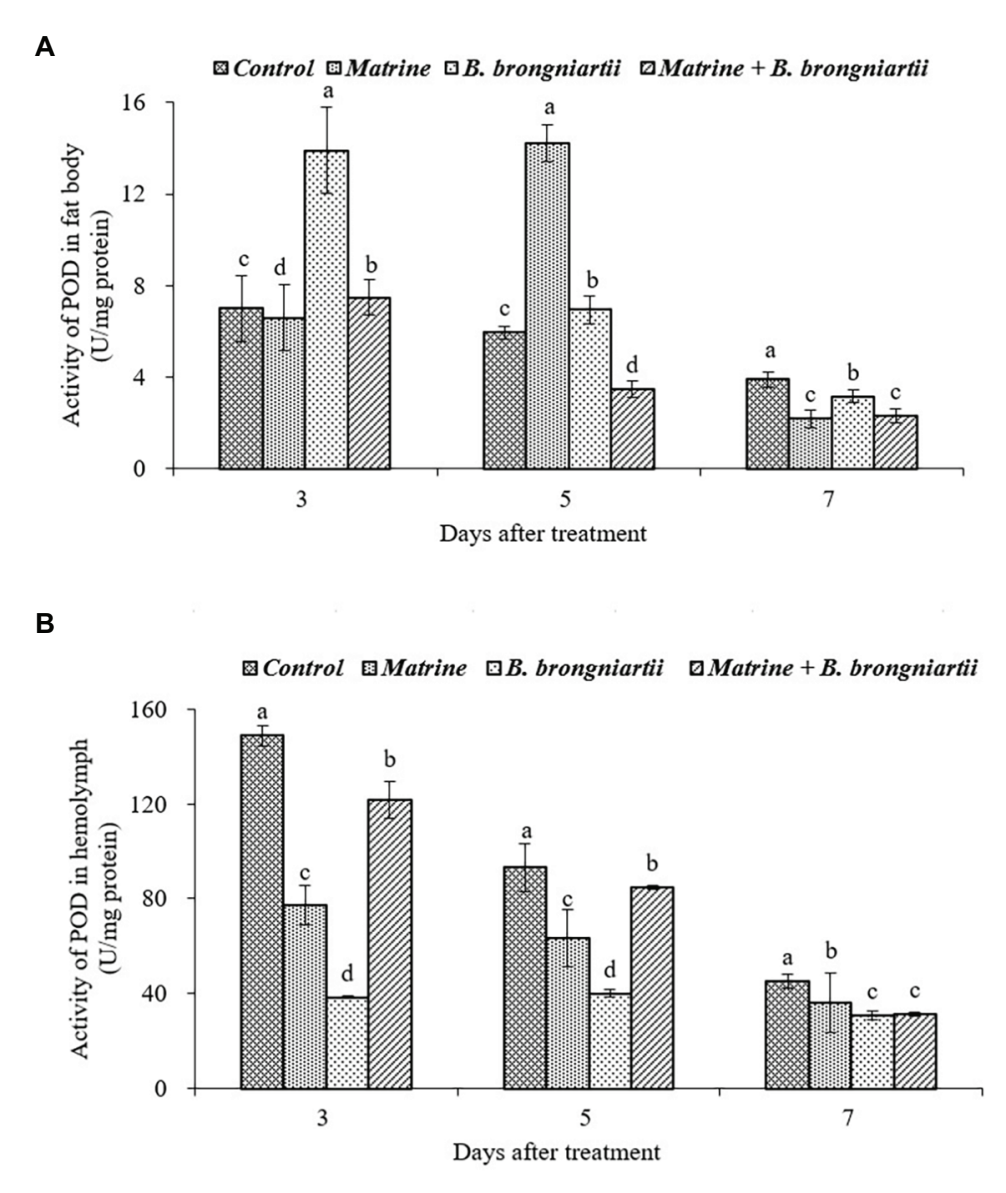
Changes in peroxidase (POD) activity of *Spodoptera litura* following treatment with *Beauveria brongniartii* and matrine **(A)** in the fat body and **(B)** in hemolymph. Bars having different letters show significant differences between treatments at different time intervals.

The exposure of the fourth instar larvae to the treated diet also resulted in varied values of POD both in the fat body and in the hemolymph (Figure 7). In the fat body, the highest activity was found on the 5th day after treatment by matrine and the lowest activity was noted on the 7th day after combined treatment by *B. brongniartii* and matrine. The values decreased gradually and became lowest in all treatments on the 7th day following treatment (Figure 7A). In the hemolymph, POD activity also showed a decreasing trend. On the 3rd and 5th days after treatment, POD activity in the hemolymph decreased when feed *B. brongniartii* and matrine separately, which was significantly lower than the combined treatment (Figure 7B).

## Discussion

Development of integrated control programs against *S.litura* involving *B. brongniartii* and chemical pesticides requires a clear understanding concerning the effects of chemicals on the physiology and pathogenicity of entomopathogenic fungi. Previous studies on *B. brongniartii* have only explained the pathogenic ability as well as lethal and sub lethal effects of this fungus against different insect pests (Fan et al., 2013; Goble et al., 2015; Mayerhofer et al., 2015; Soni et al., 2017). However, very few studies have elaborated the possible compatibility of *B. brongniartii* with natural chemicals (derived from plant or any other living organism) and other chemicals with novel mode of action (Sushil et al., 2018; Wu et al., 2019). Our previous findings revealed the synergistic interaction of *B. brongniartii* with matrine (plant derived chemical) against *S. litura* under laboratory as well as semi field conditions (Wu et al., 2019). This study reports the biological impacts of *B. brongniartii* and matrine on nutritional efficiency indices and detoxification enzymes (GST, AChE, SOD, CAT, and POD) in *S. litura.*

In the present study, the individual or joint applications of *B. brongniartii* and matrine caused reduction in nutritional efficiency indices (RGR, RCR, and ECI) of *S. litura* when compared with control although the nutritional efficiency indices (RGR, RCR, and ECI) observed for individual treatments of *B. brongniartii* and matrine were higher than those observed for joint applications of *B. brongniartii* and matrine. These results are in line with the findings of Rizwan-ul-Haq et al. (2009) who observed similar changes in RGR, RCR, and ECI of *S. exigua* in response to individual or joint treatments of ricinine (a botanical extract) and Bt (microbial pesticide). The reduction in RGR, RCR, and ECI of *S. litura* in response to *B. brongniartii* application alone or in combination with matrine can also be related to the degradation of insect gut peritrophic membrane by chitinase. Brandt et al. (1978) proposed that chitinases cause perforations in the membranes, thus facilitating entry of the pathogens into the tissues of susceptible insects. The addition of exogenous chitinase from *Streptomyces griseus* to the blood meal of the mosquito, *Anopheles freeborni*, prevented the peritrophic membrane from forming (Shahabuddin et al., 1993). Perforation of peritrophic membranes occurred *in vivo* after *Spodoptera* fifth instar larvae were fed on a diet containing recombinant ChiAII, a recombinant endochitinase encoded by *Serratia marcescens* (Regev et al., 1996).

Lower RGR, RCR, and ECI probably lead to a delay in larval growth, and the resulting formation of smaller pupa will have a direct relation to fecundity and longevity of the adult insect and even make them more susceptible to diseases and natural enemies (Khosravi et al., 2010). The rates of successful pupation as well as adult emergence of *S. litura* observed during this study were 0 when treated with *B. brongniartii* SB010 and 1.0 mg/ml matrine solution. Farooq and Freed (2016) studied the effect of a mixture of *B. bassiana* and six different insecticides, such as acetamiprid, methylamino avermectin, imidacloprid, and lufenuron, on the mortality of *Musca domestica*. Their results revealed that the lifespan, fecundity, egg hatching rate, percentage of pupation, pupal weight, and adult emergence rate of houseflies were significantly reduced, while the larval and pupal stages were prolonged.

GST is one of the main enzymes responsible for detoxification of insecticides or pathogens by insects (Claudianoc et al., 2006). Our findings showed an increase in GSTs activities during the initial 3 days following matrine treatment. This increase in enzyme activity in response shows the possible involvement of GSTs enzymes in the insecticide detoxification mechanism of *S.litura* (Jia et al., 2016). Luo and Zhang (2003) also observed similar fluctuation of GSTs in *Plutella xylostella* in response to matrine treatment. Although few reports are available on changes in GSTs activity following matrine treatment, the extent of changes can vary with change in the insect species targeted and the concentration of matrine used. In this study, we found that GSTs increased during initial periods following *B. brongniartii* treatment, which is similar to the findings of Ali et al. (2017) who also observed similar changes in GSTs activity of *B. tabaci* following *Lecanicillium muscarium* applications. They observed increases in activity of the said enzymes until 96 h post fungal application after which enzyme activities were restrained leading to metabolic imbalance and insect death. The treatment of *S.litura* with a combination of matrine and *B. brongniartii* resulted in a significant reduction of GSTs activities throughout the experimental period post 72 h of application. These results are in line with the findings of Ali et al. (2017) who have shown a similar reduction of enzyme activity in *B. tabaci* following joint application of matrine and *L. muscarium*. The changes in activities of detoxifying enzymes explained above can make the target pest more susceptible to fungal infection (Hall, 1963). The reduction in GSTs activities in response to joint application of matrine and *B. brongniartii* can be related to the sequence of chemical’s/pathogen’s action against *B. tabaci*. Insecticides normally act as a stressor increasing the susceptibility of a target pest to an entomopathogenic fungus (Furlong and Groden, 2001).

The enzyme AchE rapidly terminates nerve impulses by catalyzing the hydrolysis of the neurotransmitter, Ach at peripheral and central synapses of the insects’ nervous system (Bourne et al., 2016). AchE is also an important target site for insecticide action in the central nervous system of insects (Nathan et al., 2008). During this study, AchE activities of *S. litura* decreased throughout the experimental period following treatment with matrine and *B. brongniartii* alone or in combination. These results are in line with the findings of Ali et al. (2017) who have shown a similar reduction of AchE activities in *B. tabaci* following individual or joint application of matrine and *L. muscarium*. The decrease in AchE activities can be related to the mode of action of both agents. Matrine, an alkaloid extracted from *S. flavescens* is known to target insect Ach receptors which in turn effects AchE production (Liu et al., 2008). Luo et al. (1997) observed similar inhibition of AchE activity in turnip aphid (*L. erysimi*) following matrine application. The reduction in AchE activity of *S. litura* following *B. brongniartii* application is in line with the findings of Zibaee et al. (2009) who observed similar inhibition of AchE activity when *Beauveria bassiana* and its secondary metabolites were applied against sunn pest (*Eurygaster integriceps*). These changes in AchE activity of *S. litura* can be related to the production of a secondary metabolite named bassianolide by different species of *Beauveria*. Bassianolide can inhibit Ach receptors of insect muscles reducing the production of AchE (Xu et al., 2009).

Antioxidant enzymes (SOD, CAT, and POD) are also known as protective cellular system because SOD is known to increase the production of H_2_O_2_ from O_2_ through dismutation, while CAT and POD can catalyze H_2_O production from H_2_O_2_. These reactions can reduce the bio-membrane damage through reactive oxygen species (ROS; Ding et al., 2001). In our study, the activities of antioxidant enzymes (SOD, CAT, and POD) increased during the initial 3 days followed by a decrease when treated with *B. brongniartii* alone or in combination with matrine. The decreased activities of antioxidant enzymes (SOD, CAT, and POD) during the later experimental period can result in a reduced elimination of ROS which in turn can denature different biomolecules of the insect body. The denaturation of biomolecules can stop all the cellular processes, so leading to the death of the insect (Felton and Summers, 1995).

## Concluding Remarks

In summary, our results showed a high antifeedant activity of *B. brongniartii* and matrine on *S. litura*. The results suggest that combined treatments of matrine and *B. brongniartii* cause significant reduction in pupation (%) and adult emergence (%) of *S. litura*. The enzymatic response of *S. litura* to combined matrine and *B. brongniartii* treatment displayed a reduction in GSTs, SOD, POD, and CAT after 3 days of infection. The changes in enzymatic activities suggest that the probability of *B. brongniartii* infection was increased by matrine and *B. brongniartii* which in turn enervated the insect defense against matrine. The chemical basis of this strong synergistic effect is possibly related to the disturbance of the Ach balance and changes in AchE activities of the *S. litura* as both matrine and *B. brongniartii* can target insect Ach receptors which in turn effects AchE production. Therefore, our results have revealed the complex biochemical processes involved in the synergistic action of matrine and *B. brongniartii* against *S. litura*. These findings can provide baseline information to explain the complex biochemical processes involved in the synergistic action of matrine and *B. brongniartii* against *S. litura*.

## Data Availability Statement

The raw data supporting the conclusions of this article will be made available by the authors, without undue reservation.

## Author Contributions

JhW and SA conceived and designed the research. CZ and XtY conducted the experiments and performed the data analysis. CZ, JyL, and SA wrote the manuscript. AGSC revised the manuscript. All authors read and approved the manuscript.

### Conflict of Interest

The authors declare that the research was conducted in the absence of any commercial or financial relationships that could be construed as a potential conflict of interest.

## References

[ref1] Ahmad M. Mehmood R. (2015). Monitoring of resistance to new chemistry insecticides in *Spodoptera litura* (Lepidoptera, Noctuidae) in Pakistan. J. Econ. Entomol. 108, 1279–1288. 10.1093/jee/tov085, PMID: 26470256

[ref2] Ali S. Zhang C. Wang Z. Q. Wang X. M. Wu J. H. Cuthbertson A. G. S. (2017). Toxicological and biochemical basis of synergism between the entomopathogenic fungus *Lecanicillium muscarium* and the insecticide matrine against *Bemisia tabaci* (Gennadius). Sci. Rep. 7:46558. 10.1038/srep46558 28425450 PMC5397844

[ref3] Beauchamp C. Fridovich I. (1971). Superoxide dismutase: improved assays and an assay applicable to acrylamide gels. Anal. Biochem. 44, 276–287. PMID: 4943714 10.1016/0003-2697(71)90370-8

[ref4] Beers R. F. Sizer I. W. (1952). A spectrophotometric method for measuring the breakdown of hydrogen peroxide by catalase. J. Biol. Chem. 195, 133–140. PMID: 14938361

[ref5] Borja M. R. Franco A. W. G. Leyva E. R. Ortega C. S. Panduro A. P. (2018). Interaction of *Beauveria bassiana* and *Metarhizium anisopliae* with chlorpyrifos ethyl and spinosid in *Spodoptera frugiperda* larvae. Pest Manag. Sci. 74, 2047–2052. 10.1002/ps.4884, PMID: 29512934

[ref68] Bradford M. M. (1976). A rapid and sensitive method for the quantitation of microgram quantities of protein utilizing the principle of proteindye binding. Anal. Biochem. 72, 248–254. 10.1016/0003-2697(76)90527-3 942051

[ref6] Brandt C. R. Adang M. J. Spence K. D. (1978). The peritrophic membrane: ultrastructural analysis and function as a mechanical barrier to microbial infection in *Orgyia pseudotsugata*. J. Invertebr. Pathol. 32, 12–24.

[ref7] Bourne Y. Sharpless K. B. Taylor P. Marchot P. (2016). Steric and dynamic parameters influencing in situ cyclo additions to form triazole inhibitors with crystalline acetylcholinestrase. J. Amer. Chem. Soc. 138, 1611–1621. 10.1021/jacs.5b11384, PMID: 26731630

[ref8] Campa-Córdova A. I. Hernández-Saavedra N. Y. Ascencio F. (2002). Superoxide dismutase as modulator of immune function in American white shrimp (*Litopenaeus vannamei*). Comp. Biochem. Physiol. C Pharmacol. Toxicol. Endocrinol. 133, 557–565. 10.1016/s1532-0456(02)00125-4, PMID: 12458183

[ref9] Cerenius L. Söderhäll K. (2004). The prophenoloxidase-activating system in invertebrates. Immunol. Rev. 198, 116–126. 10.1111/j.0105-2896.2004.00116.x, PMID: 15199959

[ref10] Claudianoc C. Ranson H. Johnson R. M. Biswas S. Schuler M. A. Berenbaum M. R.. (2006). A deficit of detoxification enzymes: pesticide sensitivity and environmental response in the honeybee. Insect Mol. Biol. 15, 615–636. 10.1111/j.1365-2583.2006.00672.x, PMID: 17069637 PMC1761136

[ref11] Chaudhari C. S. Chandele A. G. Pokharkar D. S. Dethe M. D. Firake D. M. (2016). Pathogenicity of different isolates of entomopathogenic fungus, *Nomuraea rileyi* (Farlow) Samson against tobacco caterpillar, *Spodoptera litura* (Fabricius). Proc. Natl. Acad. Sci. India Sect. B Biol. Sci. 86, 1001–1007. 10.1007/s40011-015-0537-6

[ref12] Cheng X. Ye J. He H. Liu Z. Xu C. Wu B.. (2018). Synthesis, characterization and in vitro biological evaluation of two matrine derivatives. Sci. Rep. 8:15686. 10.1038/s41598-018-33908-8, PMID: 30356148 PMC6200782

[ref13] Cuthbertson A. G. S. Murchie A. K. (2005). European red spider mite—an environmental consequence of persistent chemical pesticide application. Int. J. Environ. Sci. Technol. 2, 287–290. 10.1007/BF03325888

[ref67] David W. A. L. Ellaby S. Taylor G. (1975). Rearing *Spodoptera exempta* on semi-synthetic diets and on growing maize. Entomol. Exp. Appl. 19, 226–236. 10.1111/j.1570-7458.1975.tb02374.x

[ref14] Dhar S. Jindal V. Jaryal M. Gupta V. K. (2019). Molecular characterization of new isolates of the entomopathogenic fungus *Beauveria bassiana* and their efficacy against the tobacco caterpillar, *Spodoptera litura* (Fabricius) (Lepidoptera: Noctuidae). Egypt. J. Biol. Pest Control 29:8. 10.1186/s41938-019-0110-3

[ref15] Ding S. Y. Li H. Y. Li X. F. Zhang Z. Y. (2001). Effects of two kinds of transgenic poplar on protective enzymes system in the midgut of larvae of American white moth. J. For. Res. Jpn. 12, 119–122. 10.1007/BF02867209

[ref16] Dubovskiy I. M. Slyamova N. D. Kryukov V. Y. Yaroslavtseva O. N. Levchenko M. V. Belgibaeva A. B. (2012). The activity of nonspecific esterases and glutathione-S-transferase in *Locusta migratoria* larvae infected with the fungus Metarhizium anisopliae (Ascomycota, Hypocreales). Entomol. Rev. 92, 27–31. 10.1134/S0013873812010022

[ref17] Ellman G. L. Coutney K. D. Andre V. Featherstone R. M. (1961). A new and rapid calorimetric determination of acetylcholinestrase activity. Biochem. Pharmacol. 7, 88–95. 10.1016/0006-2952(61)90145-9, PMID: 13726518

[ref18] Enayati A. A. Ranson H. Hemingway J. (2005). Insect glutathione transferases and insecticide resistance. Insect Mol. Biol. 14, 3–8. 10.1111/j.1365-2583.2004.00529.x, PMID: 15663770

[ref19] Fan J. H. Xie Y. P. Xue J. L. Zhang Y. L. Liu R. (2013). The effect of *Beauveria brongniartii* and its secondary metabolites on the detoxification enzymes of the pine caterpillar *Dendrolimus tabulaeformis*. J. Insect Sci. 13, 1–13. 10.1673/031.013.4401, PMID: 23909949 PMC3740923

[ref20] Farooq M. Freed S. (2016). Infectivity of housefly, *Musca domestica* (Diptera: Muscidae) to different entomopathogenic fungi. Braz. J. Microbiol. 47, 807–816. 10.1016/j.bjm.2016.06.002, PMID: 27522925 PMC5052330

[ref21] Felton G. W. Summers C. B. (1995). Antioxidant systems in insects. Arch. Insect Biochem. 29, 187–197. 10.1002/arch.940290208, PMID: 7606043

[ref22] Feyereisen R. (2005). “Insect cytochrome P450 in comprehensive molecular insect science.” Vol. 4. eds. Gilbert L. I. Iatrou K. Gill S. S. (Elsevier), 1–77.

[ref23] Furlong M. J. Groden E. (2001). Evaluation of synergistic interactions between the Colorado potato beetle (Coleoptera: Chrysomelidae) pathogen *Beauveria bassiana* and the insecticides, imidacloprid, and cyromazine. J. Econ. Entomol. 94, 344–356. 10.1603/0022-0493-94.2.344, PMID: 11332824

[ref24] Goble T. A. Conlong D. E. Hill M. P. (2015). Virulence of *Beauveria brongniartii* and *B. bassiana* against *Schizonycha affinis* white grubs and adults (Coleoptera: Scarabaeidae). J. Appl. Entomol. 139, 134–145. 10.1111/jen.12182

[ref25] Gopalakrishnan S. Chen F. -Y. Thilagam H. Qiao K. Xu W. -F. Wang K. -J. (2011). Modulation and interaction of immune-associated parameters with antioxidant in the immunocytes of crab *Scylla paramamosain* challenged with lipopolysaccharides. Evid.-Based Compl. Alt. 1, 1–8. 10.1155/2011/824962, PMID: 21716691 PMC3118543

[ref26] Habig W. H. (1981). “Assays for differentiation of glutathione S-transferase” in Method in enzymology. ed. Willian B. J. (New York: Academic Press), 398–405. 10.1016/s0076-6879(81)77053-8 7329316

[ref27] Hall I. M. (1963). “Microbial control” in Insect pathology an advanced treatise. ed. Steinhaus E. (New York: Academic Press), 477–511.

[ref29] Huang L. P. (2019). Identification of 29 Beauveria isolates and their virulence against *Bemisia tabaci*. M.Sc thesis. Guangzhou, China: South China Agricultural University (in Chinese).

[ref28] Huang J. Xu H. (2016). Matrine: bioactivities and structural modifications. Curr. Top. Med. Chem. 16, 3365–3378. 10.2174/1568026616666160506131012, PMID: 27150374

[ref30] Ishtiaq M. Saleem M. A. Razaq M. (2012). Monitoring of resistance in *Spodoptera exigua* (Lepidoptera: Noctuidae) from four districts of the southern Punjab, Pakistan to four conventional and six new chemistry insecticides. Crop Prot. 33, 13–20. 10.1016/j.cropro.2011.11.014

[ref31] Jia M. Cao M. Li Y. Tu X. Wang G. Nong X.. (2016). Biochemical basis of synergism between pathogenic fungus *Metarhizium anisopliae* and insecticide chlorantraniliprole in *Locusta migratoria* (Meyen). Sci. Rep. 6:28424. 10.1038/srep28424, PMID: 27328936 PMC4916465

[ref32] Khosravi R. Sendi J. J. Ghadamyari M. (2010). Effect of *Artemisia annua* L. on deterrence and nutritional efficiency of lesser mulberry pyralid (*Glyphodes pylolais* Walker) (Lepidoptera: Pyralidae). J. Plant Prot. Res. 50, 423–428. 10.2478/v10045-010-0071-8

[ref70] Koul O. Kaur H. Goomber S. Wahab S. (2004). Bio-efficacy and mode of action of rocaglamide from *Aglaia elaeagnoidea* (Syn. *A. roxburghiana*) against gram pod borer, Helicoverpa armigera (Hubner). J. Appl. Entomol. 128, 177–184. 10.1111/j.1439-0418.2004.00829.x

[ref69] Koul O. Shankar J. S. Mehta N. Taneja S. C. Tripathi A. K. Dhar K. L. (1997). Bio-efficacy of crude extracts of *Aglaia species* (Meliaceae) and some active fractions against lepidopteran larvae. J. Appl. Entomol. 121, 245–248. 10.1111/j.1439-0418.1997.tb01400.x

[ref34] Li S. G. Hau R. M. Lin H. F. Cao H. Q. Hu J. Hu P. (2010). Control effects of four biological pesticides and two chemical pesticides and their mixtures against mixed population of *Nilaparvata lugens* and *Sogatella furcifera*. Chin. Bull. Entomol. 47, 768–772.

[ref33] Li X. Schuler M. A. Berenbaum M. R. (2007). Molecular mechanisms of metabolic resistance to synthetic and natural xenobiotics. Annu. Rev. Entomol. 52, 231–253. 10.1146/annurev.ento.51.110104.151104, PMID: 16925478

[ref39] Liu L. J. Alam M. S. Hirata K. Matsuda K. Ozoe Y. (2008). Actions of quinolizidine alkaloids on *Periplanta Americana* nicotinic acetylcholine receptors. Pest Manag. Sci. 64, 1222–1228. 10.1002/ps.1622, PMID: 18566954

[ref35] Liu S. Niu H. Xiao T. Xue C. Liu Z. Luo W. (2009). Does phenoloxidase contributed to the resistance? Selection with butane-fipronil enhanced its activities from diamondback moths. Open Biochem. J. 3, 9–13. 10.2174/1874091X00903010009, PMID: 19401784 PMC2674291

[ref37] Liu C. H. Wang Y. W. Yu H. C. Sun Y. F. Hou Y. M. Zhao K. J. (2016). Toxicity of *Beauveria brongniartii* to two cutworm species. Chin. J. Appl. Entomol. 53, 739–744.

[ref36] Liu Y. Xu Y. Ji W. Li X. Sun B. Gao Q.. (2014). Anti-tumor activities of matrine and oxymatrine: literature review. Tumor Biol. 35, 5111–5119. 10.1007/s13277-014-1680-z, PMID: 24526416

[ref38] Lumjuan N. McCarroll L. Prapanthadara L. Hemingway J. Ranson H. (2005). Elevated activity of an epsilon class glutathione transferase confers DDT resistance in the dengue vector, *Aedes aegypti*. Insect Biochem. Mol. Biol. 35, 861–871. 10.1016/j.ibmb.2005.03.008, PMID: 15944082

[ref40] Luo W. C. Li Y. S. Mu L. Y. (1997). The toxicities of alkaloids from *Sophora alopecuroides* against turnip aphids and effects on several estereases. Acta Entomol. Sin. 40, 358–365 (in Chinese).

[ref41] Luo W. C. Zhang Q. (2003). The effects of *Sophora alopecuroids* alkaloids on metabolic esterases of the diamondback moth. Acta Entomol. Sin. 46, 122–125.

[ref42] Mao L. X. Henderson G. (2007). Antifeedant activity and acute and residual toxicity of alkaloids from *Sophora flavescens* (Leguminosae) against formosan subterranean termites (Isoptera: Rhinotermitidae). J. Econ. Entomol. 100, 866–870. 10.1603/0022-0493(2007)100[866:aaaaar]2.0.co;2, PMID: 17598549

[ref43] Mayerhofer J. Enkerli J. Zelger R. Strasser H. (2015). Biological control of the European cockchafer: persistence of *Beauveria brongniartii* after long term application in the euro region Tyrol. BioControl 60, 617–629. 10.1007/s10526-015-9671-6

[ref44] Müller P. Donnelly M. J. Ranson H. (2007). Transcription profiling of a recently colonised pyrethroid resistant *Anopheles gambiae* strain from Ghana. BMC Genomics 8:36. 10.1186/1471-2164-8-36, PMID: 17261191 PMC1797171

[ref55] Nathan S. S. Choi M. Y. Seoa H. Y. Paik C. H. Kalaivani K. Kim J. D. (2008). Effect of azadirachtin on acetylcholinesterase (AChE) activity and histology of the brown planthopper *Nilaparvata lugens* (Stål). Ecotox. Environ. Safe. 70, 244–250. 10.1016/j.ecoenv.2007.07.005, PMID: 17765967

[ref45] Nguyen H. C. Tran T. V. Ngyuen Q. L. Nguyen N. N. Nguyen M. K. Nguyen N. T. T. (2017). Newly isolated *Paecilomyces lilacinus* and *paecilomyces javanicus* as novel biocontrol agents for *Plutella xylostella* and *Spodoptera litura*. Notu. Bot. Hort. Agrobot. Clujnapoca. 45, 280–286. 10.15835/nbha45110726

[ref46] Oehmichen M. Besserer K. (1982). Forensic significance of acetylcholine esterase histochemistry in organophosphate intoxication. Int. J. Leg. Med. 89, 149–165. 10.1007/BF01873797, PMID: 6760603

[ref47] Ortelli F. Rossiter L. C. Vontas J. Ranson H. Hemingway J. (2003). Heterologous expression of four glutathione transferase genes genetically linked to a major insecticide-resistance locus from the malaria vector *Anopheles gambiae*. Biochem. J. 373, 957–963. 10.1042/BJ20030169, PMID: 12718742 PMC1223529

[ref48] Ou D. (2019). Toxicity evaluation of entomopathogenic fungi against Asian citrus psyllid. M.Sc thesis. Guangzhou, China: South China Agricultural University (in Chinese).

[ref52] Regev A. Keller M. Strizhov N. Sneh B. Prudovsky E. Chet I.. (1996). Synergistic activity of a *Bacillus thuringiensis* delta-endotoxin and a bacterial endochitinase against *Spodoptera littoralis* larvae. Appl. Environ. Microbiol. 62, 3581–3586. PMID: 8837413 10.1128/aem.62.10.3581-3586.1996 PMC168163

[ref49] Revathi K. Chandrasekaran R. Thanigaivel A. Kirubakaran S. A. Nathan S. S. (2014). Biocontrol efficacy of protoplast fusants between *Bacillus thuringiensis* and *Bacillus subtilis* against *Spodoptera litura* Fabr. Arch. Phytopathol. Plant Prot. 47, 1365–1375. 10.1080/03235408.2013.840999

[ref53] Rizwan-ul-Haq M. Hu Q. B. Hu M. Y. Lin Q. S. Wang Q. L. (2009). Biological impacts of harmaline, ricinine and their combined effects with *Bacillus thuringiensis* on *Spodoptera exigua* (Lepidopter: Noctuidae). J. Pestic. Sci. 82, 327–334. 10.1007/s10340-009-0257-x

[ref54] Roslavtseva S. A. Bakanova E. I. Eremina O. Y. (1993). Esterases in arthropods and their role in the mechanisms of insect acaricide detoxification. Izv. Akad. Nauk Biol. 3, 368–375.

[ref50] Serebrov V. V. Gerber O. N. Malyarchuk A. A. Martemyanov V. V. Aleksee A. A. Glupov V. V. (2006). Effect of entomopathogenic fungi on detoxification enzyme activity in greater wax moth *Galleria mellonella* L. (Lepidoptera, Pyralidae) and role of detoxification enzymes in development of insect resistance to entomopathogenic fungi. Biol. Bull. 33, 581–586. 10.1134/S1062359006060082

[ref56] Shahabuddin M. Toyoshima T. Aikawa M. Kaslow D. C. (1993). Transmission-blocking activity of a chitinase inhibitor and activation of malarial parasite chitinase by mosquito protease. Proc. Natl. Acad. Sci. U. S. A. 90, 4266–4270. 10.1073/pnas.90.9.4266, PMID: 8483942 PMC46487

[ref51] Shannon L. M. Kay E. Lew J. Y. (1966). Peroxidase isozymes from horseradish roots. I. Isolation and physical properties. J. Biol. Chem. 241, 2166–2172. PMID: 5946638

[ref57] Soni S. Mehta P. K. Chandel R. S. (2017). Susceptibility of white grub, *Brahmina coriacea* (Hope) infesting potato to local strains of *Beauveria brongniartii* (Saccardo) in Himachal Pradesh. J. Biol. Control 32, 41–47. 10.18311/jbc/2018/16288

[ref58] Sushil S. N. Josh D. Tripathi G. M. Singh M. R. Baitha A. Rajak D. C. (2018). Exploring efficacious microbial bio-agents and insecticides against white grubs in sugarcane in indo-gangetic plains. Sugar Tech. 20, 552–557. 10.1007/s12355-017-0556-0

[ref59] Terriere L. C. (1984). Induction of detoxication enzymes in insects. Annu. Rev. Entomol. 29, 71–88. 10.1146/annurev.en.29.010184.000443, PMID: 6362551

[ref61] Wu J. H. Yu X. Wang X. S. Tang L. D. Ali S. (2019). Matrine enhances the pathogenicity of *Beauveria brongniartii* against *Spodoptera litura* (Lepidoptera: Noctuidae). Front. Microbiol. 10:1812. 10.3389/fmicb.2019.01812, PMID: 31456766 PMC6700297

[ref60] Wu Q. J. Zhang Y. J. Xu B. Y. Zhang W. J. (2011). The defending enzymes in abamectin resistant *Plutella xylostella*. Chinese J. Appl. Entomol. 48, 291–295.

[ref62] Xu Y. Q. Orozco R. Wijerante E. M. K. Espinosa-Artiles P. Lesile Gunatilaka A. A. Stock S. P.. (2009). Biosynthesis of the cyclooligomer depsipeptide bassianolide, an insecticidal virulence factor of *Beauveria bassiana*. Fungal Genet. Biol. 46, 353–364. 10.1016/j.fgb.2009.03.001, PMID: 19285149

[ref63] Yang H. Qin C. S. Chen Y. M. Zhang G. Y. Dong L. H. Wan S. Q. (2019). Persistence of *Metarhizium* (Hypocreales: Clavicipitaceae) and *Beauveria bassiana* (Hypocreales: Clavicipitaceae) in tobacco soils and potential as biocontrol agents of *Spodoptera litura* (Lepidoptera: Noctuidae). Environ. Entomol. 48, 147–155. 10.1093/ee/nvy161, PMID: 30508198

[ref64] Zanardi O. Z. Ribeiro L. D. P. Ansante T. F. Santos M. S. Bordini G. P. Yamamotoa P. T. (2015). Bioactivity of a matrine-based biopesticide against four pest species of agricultural importance. Crop Prot. 67, 160–167. 10.1016/j.cropro.2014.10.010

[ref65] Zhang S. H. Zhang Y. Zhuang Y. Han Y. X. (2012). Matrine induces apoptosis in human acute myeloid leukemia cells via the mitochondrial pathway and Akt inactivation. PLoS One 7:e46853. 10.1371/journal.pone.0046853, PMID: 23056487 PMC3466205

[ref66] Zibaee A. Bandani A. R. Tork M. (2009). Effect of the entomopathogenic fungus, Beauveria bassiana, and its secondary metabolite on detoxifying enzyme activities and acetylcholinesterase (AChE) of the Sunn pest, *Eurygaster integriceps* (Heteroptera: Scutellaridae). Biocontrol Sci. Tech. 19, 485–498. 10.1080/09583150902847127

